# When treatment success is not as black and white as a radiograph: the bio/psycho/social impact of chronic osteomyelitis

**DOI:** 10.11604/pamj.2014.19.170.5011

**Published:** 2014-10-20

**Authors:** Freya Jane Yoward, Sarah Lucy Bury

**Affiliations:** 1 School of Medicine and Dentistry, Bristol University, Bristol, England

**Keywords:** Osteomyelitis, bone deformity, disability, Malawi

## Abstract

This case report is from Malawi Beit CURE international Hospital (BCIH), a center of excellence for the management of chronic osteomyelitis (COM) in children. Currently minimal evidence based data exists on the long term outcomes for treatment of bone defects following COM. Few studies evaluating outcomes are based solely on clinical parameters. This case study highlights the often-debilitating outcome after treatment and hence the need for further research to find the most effective treatment to create treatment guidelines. It particularly demonstrates the importance of the bio/psycho/social impact of long-term morbidity and disability, which must be considered alongside clinical outcomes in evaluation.

## Introduction

Chronic osteomyelitis (COM) is defined as bone infection and inflammation lasting longer than 3 months [1]. COM commonly causes long-term defects and morbidity to children; the burden of which is often greater in developing countries due to, poor recognition, late presentation and incomplete treatment. Where treatment is available surgery is needed to eradicate infection and correct deformities. Due to the global distribution of the disease there is minimal research comparing long-term outcomes after treatment for COM, particularly treatment of bone defects. Consequently, there are no published guidelines or protocols for treatment. Treatments are commonly unsuccessful; some patients require multiple revision surgeries and are left with long-term physical, psychological and socioeconomic morbidity and disability.

## Patient and observation

A 9-year-old boy named T.S, presented to BCIH for recurrent treatment of a bone defect caused by COM. T.S had a 3-year history of chronic osteomyelitis following a closed fracture of the right that which was managed conservatively by a district hospital. Subsequent development of chronic osteomyelitis progressed unnoticed for 3 months with vague bone pain and no fever.

**Figure 1:**
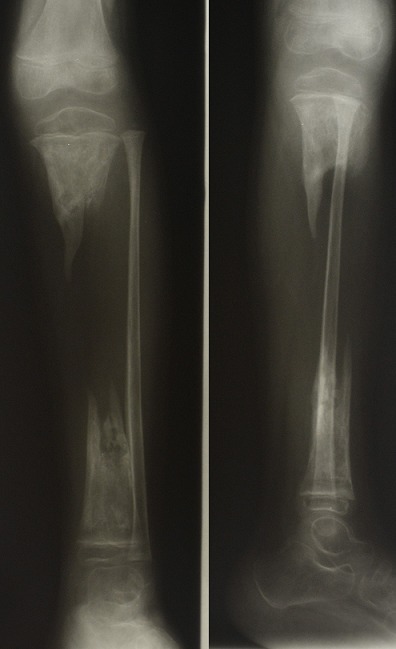
Lateral and PA radiograph of the right tibia and fibula post sequestectomy showing a large segmental bone defect

**Figure 2:**
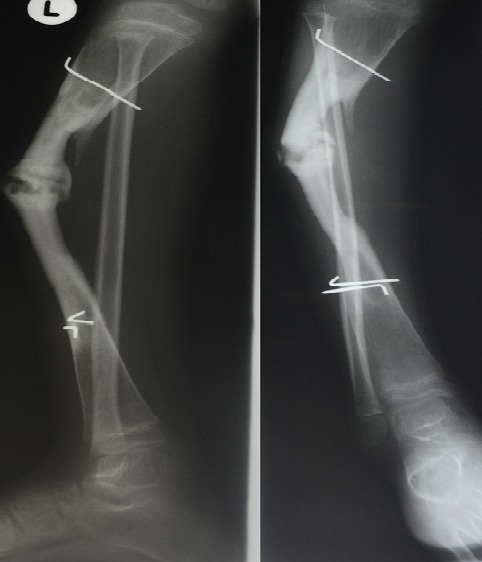
Lateral and AP radiograph of the right leg, showing a non-union fracture through a previous fibular graft in the tibia taken from the contralateral leg

**Figure 3:**
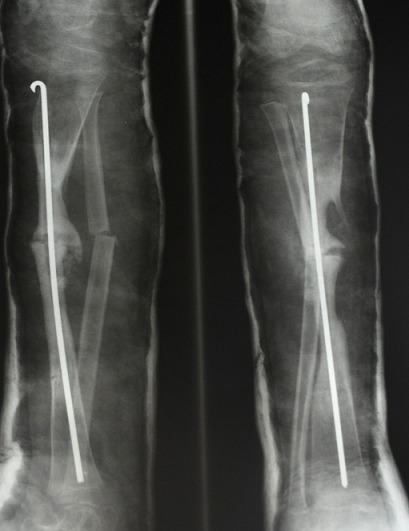
Lateral and AP radiograph of the right leg showing correction of a non-union fracture of the tibia with a rush rod

By the time access to a tertiary care was made and chronic osteomyelitis was diagnosed at BCIH, radiographs showed a large circumferential sequestrum with reduced structural integrity of the right tibia. Aggressive surgical debridement and circumferential sequestectomy was needed to attempt clearance of all infection, this resulted in a large segmental bone defect (Figure 1). Reconstructive treatment was consequentially needed using a contralateral fibular bone graft. T.S was readmitted to BCIH, 2 years on, due to a painless anterior deformity of his right leg. He had been walking with the aid of a stick since the tibia reconstruction. Radiographs showed that the graft within the right tibia had united however, there was a non-union fatigue fracture through the graft and anterior deformity of the tibia bone (Figure 2). To repair this T.S's third radical surgical treatment in 3 years was by open reduction and insertion of a rush rod (Figure 3). He was discharged for follow up once medically stable and imaging showed a stable correction.

Remarkably, a qualitative questionnaire used at 5-year review revealed that the most challenging part of T.S's condition was not the repeated unsuccessful surgical treatment, time in hospital, or general fatigue associated with a chronic condition. Instead, he suffered most from the ridicule, bullying and isolation he encountered from his peers due to his deformity and disability. Despite this, the psychosocial impact of his condition was neither documented nor known of to the medical team. During the review T.S described feeling “*different and isolated*” because he walked with the aid of a stick and couldn′t play with his friends. He said that people in his village referred to him as “*the disabled one*” and bullied him. His mother described conspiracies that her son was cursed. Importantly his attendance at school had reduced due to bullying.

## Discussion

Acquired disability from COM can be extremely debilitating to the patient yet little recognition of this is made in current available literature. In developing countries such as Malawi only 2% of children have access to rehabilitation and basic support [2]. In this case T.S had received no governmental or community based support for his disability.

In 2010 a qualitative study found that indignity and exclusion, as T.S. experienced, are extremely common outcomes for children living with musculoskeletal impairments in Malawi [3]. They also found that for the majority of people, the need for emotional support exceeded that of economic support [3]. This case report reflects the importance of these findings; in countries such as Malawi where a vast amount of practical support is needed, emotional support can be easily overlooked.

T.S. had poor school attendance due to the psychosocial impact of his condition. Less than 10% of African children living with disability are estimated to attend school due to physical and psychological barriers [3]. This finding is reiterated in findings by SINTEF report; children living with disability were almost twice as likely never to have attended school [4].

In 2011 the prevalence of disability in Malawi, was measured at 4.18%, 43% caused by a physical disability [5]. Yet WHO estimates that 15% of the world's population lives with a disability [2] indicating this percentage may be an underestimation. UNICEF reports provide a potential explanation for this underestimation, it reports that many people with disabilities in under-developed countries are excluded from research; in some cases their births even go unregistered [6].

Globally, there are strategies designed to encourage and educate for inclusion and support for people living with disability within communities [3]. The community based rehabilitation framework produced by WHO provides guidelines on possible ways to implement support. The Malawi council for the handicapped is the most successful organization currently implementing this framework in Malawi but funding is scarce. Disability leaders have been trained within districts but they struggle with the practicalities of providing support within the villages.

Recently the Malawian government produced a disability bill [7] with the aim to promote the rights of people living with disability, allowing them to play an even role within society. This will hopefully encourage more funding, more research, and more emphasis on disability and will highlight the need for holistic care with a focus on the bio/psycho/social impacts of long term diseases like COM. Ultimately however awareness, education and recognition of patients’ struggles is fundamentally important.

## Conclusion

COM is common in developing countries. Further evidence-based research into long-term outcomes comparing treatment options is needed to establish treatment guidelines. Currently, patients are commonly left with significant morbidity and disability following surgical treatment for resultant bone defects. This can restrict the child from functioning normally within their family and social community and have significant psychosocial and economical implications for the child and their family. Psychosocial outcomes must be recognized and support needs to be considered for the patient. Community education can provide a sustainable beginning for solutions to enable recognition and holistic support for those struggling to live with disability.
